# Peripheral Osteoma on the Medial Surface of the Mandibular Ramus: A Case Report

**DOI:** 10.7759/cureus.106765

**Published:** 2026-04-10

**Authors:** Mayur M Shingade, Chaitanya S Khalane, Yash Uplekar, Rushikesh Laware

**Affiliations:** 1 Department of Oral and Maxillofacial Surgery, Jawahar Medical Foundation's Annasaheb Chudaman Patil Memorial Dental College, Dhule, IND; 2 Department of Oral Pathology, Jawahar Medical Foundation's Annasaheb Chudaman Patil Memorial Dental College, Dhule, IND

**Keywords:** benign, mandible, osteoma, radiography, surgical excision

## Abstract

Peripheral osteomas are uncommon benign osteogenic lesions that arise from the periosteum, with the lingual mandibular surface being a recognized site. However, solitary pedunculated lesions at the posterior medial ramus-body junction are rare. This case report describes a 34-year-old man who presented with mild, intermittent dull pain in the right posterior mandible, exacerbated by mastication for three months, without swelling or paresthesia. Clinical examination revealed tenderness near the ramus-body junction. Cone-beam computed tomography (CBCT) demonstrated a well-circumscribed, pedunculated radiopaque mass (1.5 cm) with a homogeneous bone-like density attached via a narrow stalk to the medial surface of the right mandibular ramus-body junction, with an intact surrounding cortex and no periosteal reaction. Surgical excision via an extraoral submandibular approach allowed optimal visualization and safe en bloc removal. Histopathological examination confirmed the presence of mature lamellar bone with Haversian systems and a thin fibrous periosteal covering, lacking cellular atypia or cartilaginous elements, which is diagnostic of compact-type peripheral osteoma. Postoperative recovery was uneventful, with complete pain resolution and no recurrence at six-month follow-up. This case highlights the value of CBCT in preoperative planning and the efficacy of conservative surgical excision of symptomatic peripheral osteomas in anatomically challenging posterior mandibular locations.

## Introduction

Osteomas are benign osteogenic tumors characterized by the proliferation of mature compact and/or cancellous bone [1]. They are classified into three types: central (endosteal), peripheral (periosteal), and extraskeletal. Peripheral osteomas arise from the periosteum and present as sessile or pedunculated masses attached to the cortical surface [2,3]. Peripheral osteomas of the jaws are uncommon, with the mandible being affected more frequently than the maxilla [4]. Common sites in the mandible include the lingual aspect of the body, angle, inferior border, condyle, and ramus. The overall incidence is low, estimated at 0.01-0.04% of the population, and solitary peripheral osteomas comprise a small proportion of benign bone tumors [3,5].

Clinically, these lesions are typically slow-growing and asymptomatic and are often discovered incidentally during routine examinations or imaging [4]. When symptomatic, they may present as a hard, palpable swelling causing facial asymmetry, mild discomfort, or functional issues, such as occlusal interference or limitation in jaw movement, depending on the size and location [5,6]. Histologically, osteomas consist of dense mature lamellar bone with haversian systems in compact forms or trabecular bone with fibrous stroma in cancellous variants [7]. Osteoblastic rimming may be present, but there is no cellular atypia, increased mitotic activity, or evidence of malignancy [3,8]. The differential diagnoses include torus mandibularis, exostoses, osteochondroma, osteoblastoma, ossifying fibroma, fibrous dysplasia, condensing osteitis, cementoblastoma, and, rarely, low-grade osteosarcoma [1,4,6]. Multiple osteomas raise suspicion for Gardner syndrome, which requires systemic evaluation for associated intestinal polyposis and other features [9].

This case describes a periosteal osteoma arising from the medial (lingual) surface of the posterior mandibular body-ramus junction, which is an uncommon presentation. Although the lingual aspect of the mandibular body is among the more frequently reported sites for peripheral osteomas in the mandible, solitary lesions specifically attached to the periosteum in this exact posteromedial location remain rare, with limited well-documented cases emphasizing the value of reporting such variants to contribute to the diagnostic and management literature in oral and maxillofacial pathology.

## Case presentation

A 34-year-old male patient presented to the Department of Oral and Maxillofacial Surgery at Jawahar Medical Foundation's Annasaheb Chudaman Patil Memorial Dental College, Dhule, India, in December 2024, with a chief complaint of mild, intermittent pain in the right posterior mandible persisting for three months. The pain was dull, non-radiating, and exacerbated by mastication, without associated swelling, fever, or paresthesia. The medical history revealed no systemic illnesses, allergies, or habits such as tobacco use. The patient did not report any trauma or prior dental procedures in the region.

Clinical examination

Facial symmetry was maintained, with no palpable lymphadenopathy or skin changes. The oral mucosa appeared normal, with no erythema or ulceration. Palpation of the right posterior mandible elicited mild tenderness medially near the ramus-body junction, but no obvious swelling or crepitus was noted. The occlusion was Class I, and all teeth were vital with no caries or periodontal involvement. The range of jaw motion was normal, although slight discomfort occurred during wide opening. No neurological deficits were noted.

Radiographic examination

**Figure 1:**
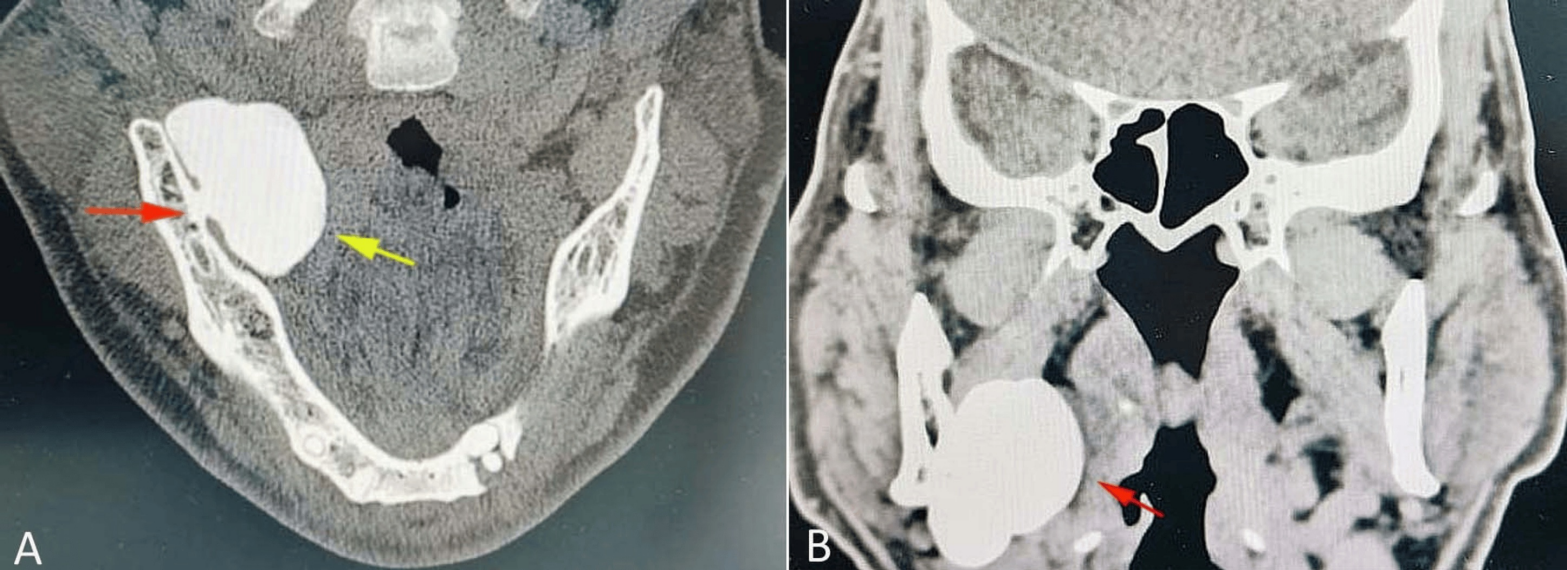
Cone-beam computed tomographic (CBCT) image of the mandible. (A) Axial view showing radiopaque lesion (yellow arrow) attached with stalk (red arrow) on the medial aspect of the right posterior mandible. (B) Coronal view showing radiopaque lesion (red arrow) on the medial surface of the right posterior mandible causing mild displacement of adjacent soft tissues but no invasion. Original CBCT image of the patient, used with patient's permission.

A CBCT scan was performed to evaluate the symptomatic area. The axial view (Figure 1A) revealed a well-circumscribed, pedunculated, radiopaque mass approximately 1.5 cm in diameter on the medial surface of the right mandibular ramus-body junction. The lesion exhibited a homogeneous bone-like density with a clear cortical outline, attached via a narrow stalk, and no involvement of adjacent soft tissues or cortical erosion (Figure 1B). The surrounding bone appeared intact, with no periosteal reaction or pathological fractures. Panoramic and intraoral radiography corroborated these findings, ruling out odontogenic origins.

Provisional diagnosis

Based on the clinical presentation and radiographic features, such as an asymptomatic, slow-growing bony exostosis in a non-tooth-bearing area, the provisional diagnosis was a peripheral osteoma of the mandible. Differential diagnoses included torus mandibularis, osteochondroma, and calcified lymph nodes; however, the pedunculated morphology and location favored osteoma. A literature review indicates that peripheral osteomas are rare benign tumors, comprising <1% of mandibular lesions, often arising from the periosteum on the lingual aspect, with a male predilection in the third decade.

Treatment protocol

**Figure 2:**
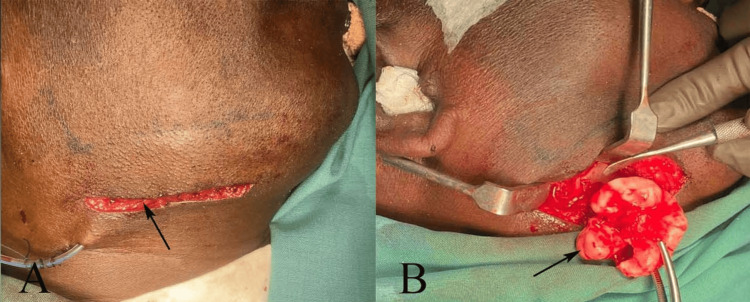
(A) Submandibular incision (black arrow) approximately 2 cm below the inferior border of the mandible on the right side. (B) Excised bony protuberance (black arrow) as a pedunculated, ivory-like mass. Original images of the patient, used with patient's permission.

Under local anesthesia, surgical excision was planned via an extraoral approach to ensure optimal visualization and safe access to the lesion on the medial surface of the mandibular ramus, given its location and the need for precise control in the vicinity of critical structures. A submandibular incision (Risdon approach) was made in a natural skin crease approximately 2 cm below the inferior border of the mandible on the right side (Figure 2A), extending approximately 5-6 cm to provide adequate exposure while minimizing the cosmetic impact.

Layered dissection was performed through the skin, platysma, and superficial cervical fascia, with careful identification and preservation of the marginal mandibular branch of the facial nerve. The pterygomasseteric sling was divided, and subperiosteal elevation was used to expose the medial aspect of the ramus-body junction. The bony protuberance was clearly visualized as a pedunculated, ivory-like mass. The lesion was osteotomized at its base using a chisel and mallet, with gentle tapping to avoid fracture propagation, and removed en bloc (Figure 2B). Hemostasis was meticulously achieved using bipolar cautery and bone wax, as needed. The surgical site was thoroughly irrigated with saline, and any residual periosteum or soft-tissue tags were excised. Primary closure was performed in layers: deep tissues with 3-0 Vicryl interrupted sutures and skin with 5-0 Prolene subcuticular sutures for optimal cosmesis. A small suction drain was placed and removed after 24 hours. Prophylactic antibiotics (amoxicillin-clavulanate 625 mg BID for five days), a reduced twice-daily regimen was selected to limit antibiotic overuse in a non-infected case, and analgesics (ibuprofen 400 mg TID with tramadol as rescue) were prescribed. Postoperative recovery was uneventful, with minimal edema, pain resolution within a week, and excellent wound healing with no facial nerve weakness or complications at the 6-month follow-up. The extraoral approach facilitated complete excision without compromising adjacent structures, aligning with literature recommendations for lesions on the medial ramus, where intraoral access may be limited.

Histopathological examination and final diagnosis

**Figure 3:**
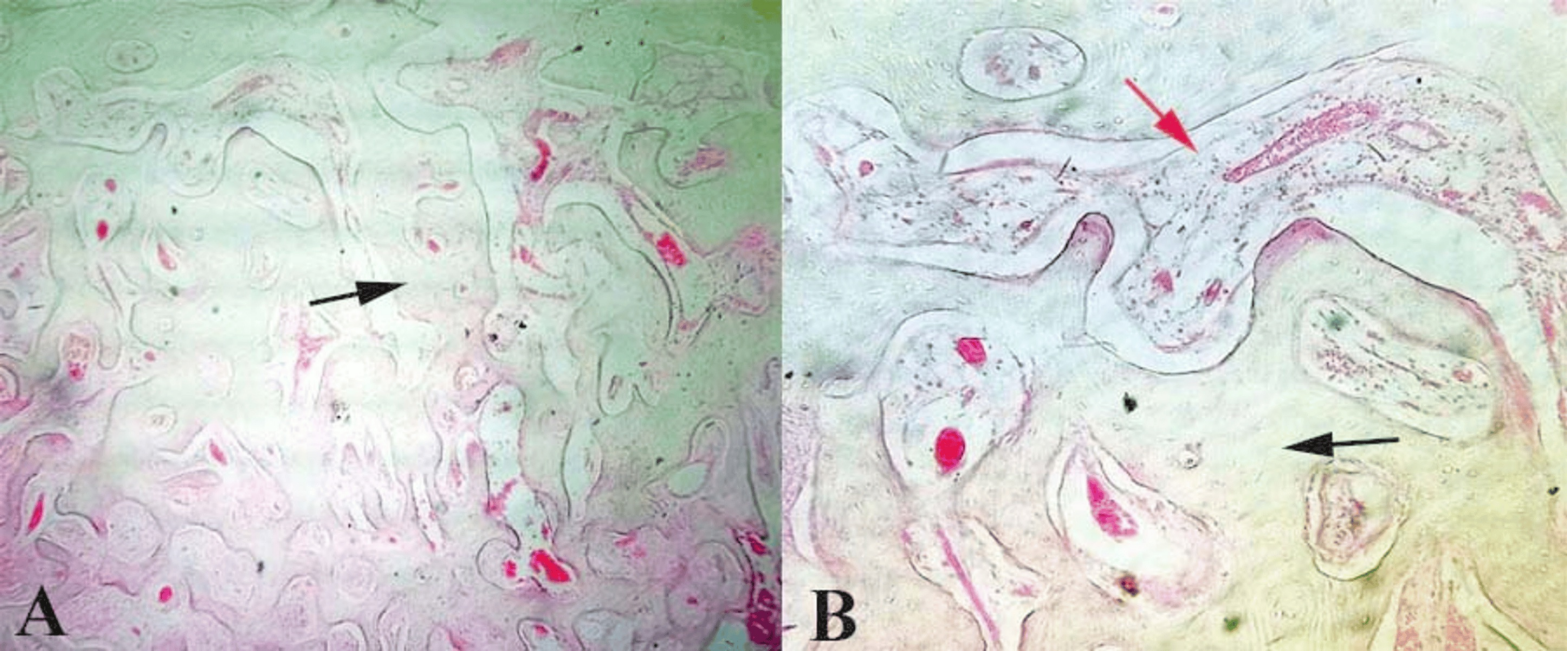
Hematoxylin and eosin-stained section of tissue specimen at (A) 10x magnification showed lamellar bone (black arrow). (B) 40x magnification showed lamellar bone without inflammation (black arrow) and fibrous marrow tissue (red arrow). Original images of the tissue sample.

The excised specimen, a firm, ivory-like mass, was submitted for histological examination. Microscopic examination revealed mature lamellar bone with Haversian systems, covered by a thin fibrous periosteum, devoid of atypical cells or inflammation. No cartilaginous or odontogenic elements were observed (Figure 3). These findings confirmed the diagnosis of peripheral osteoma, consistent with the literature describing it as a hamartomatous proliferation of compact bone. Long-term monitoring is advised to detect any syndromic associations, such as Gardner's syndrome, although it is absent here.

## Discussion

The present case of a periosteal osteoma on the medial surface of the posterior mandibular body near the ramus-body junction in a 34-year-old man aligns with the established rarity of peripheral osteomas in the mandible, which are benign, slow-growing osteogenic tumors arising from the periosteum and more commonly affecting the lingual aspect of the body, angle, inferior border, condyle, and ramus [3,4,6]. The literature consistently reports mandibular predominance over maxillary involvement, with solitary peripheral osteomas representing a small subset of benign bone tumors, typically presenting in the third to fourth decades of life and showing a slight male predilection [1,4,7]. Although the lingual aspect of the mandibular body is among the more frequently documented sites, pedunculated or sessile lesions specifically at the posterior medial ramus-body junction remain uncommon, as most reported cases cluster around the angle, condyle, anterior body, or ramus proper [10,11].

The exact etiology of peripheral osteomas remains unclear; however, several theories have been proposed. These include developmental anomalies, true neoplastic processes, and reactive mechanisms secondary to trauma, infection, or muscle traction. Minor, repetitive trauma and periosteal irritation are considered significant contributing factors, leading to localized bone proliferation. In addition, genetic associations, particularly in conditions such as Gardner’s syndrome, further support a multifactorial origin [6,9].

Clinically, the patient's mild, intermittent dull pain exacerbated by mastication, without swelling, paresthesia, or significant functional limitation, is characteristic of symptomatic peripheral osteomas, which are often incidental findings or cause subtle discomfort due to mechanical interference during mastication [3,6]. CBCT demonstrated a well-circumscribed, pedunculated radiopaque mass approximately 1.5 cm in diameter, with homogeneous bone-like density, a narrow stalk attachment, intact surrounding cortex, and absence of erosion or periosteal reaction, which are radiographic hallmarks that strongly support peripheral osteoma and differentiate it from differentials such as osteochondroma, osteoblastoma, torus mandibularis, or ossifying fibroma [7,8,12]. CBCT's superior resolution of CBCT for cortical and medullary details has proven invaluable in preoperative planning, consistent with its increasing role in the diagnosis of such lesions [4,13].

Surgical management via an extraoral submandibular (Risdon) approach enabled optimal visualization and safe excision of the medial ramus lesion, with careful preservation of the marginal mandibular branch of the facial nerve, en bloc osteotomy, and layered closure, in accordance with recommendations for posteriorly or medially located lesions, where intraoral access may be limited or riskier near vital structures [10,11,14]. Histopathological examination revealed mature lamellar bone with prominent Haversian systems, thin periosteal covering, and no atypia, inflammation, or cartilaginous/odontogenic elements, confirming the compact-type peripheral osteoma as a hamartomatous proliferation [7,8].

Recurrence following complete excision is extremely rare, with rates reported as <1-5% in the literature, and no documented cases of malignant transformation [9,10]. The uneventful postoperative course, rapid pain resolution, and absence of complications at the 6-month follow-up underscore the efficacy of conservative surgical intervention. No syndromic associations (such as Gardner syndrome) were evident, negating the need for systemic screening [9]. This case highlights the utility of CBCT in accurate diagnosis, the appropriateness of extraoral access for medial posterior lesions, and the benign prognosis of peripheral osteomas, while contributing to the limited documentation of such atypical sites to enhance diagnostic awareness and management strategies in oral and maxillofacial pathology [4,5].

## Conclusions

Peripheral osteomas of the posterior medial mandibular body near the ramus-body junction are rare but should be considered in the differential diagnosis of solitary bony protuberances in this region, particularly when patients present with mild mechanical pain. The present case highlights the critical role of CBCT in accurately characterizing lesion morphology, cortical integrity, and relationships with adjacent structures, thereby guiding appropriate surgical planning. Although the lingual aspect of the mandible is a known predilection site for peripheral osteomas, the pedunculated morphology and exact posteromedial location described here add to the limited documentation of such variants. This case reinforces the benign, hamartomatous nature of peripheral osteomas and contributes valuable clinical, radiographic, and therapeutic data to the oral and maxillofacial pathology literature, encouraging clinicians to maintain diagnostic vigilance for such rare presentations of peripheral osteomas.
